# Hydrophobic Formulations Based on Tall Oil Distillation Products for High-Density Fiberboards

**DOI:** 10.3390/ma13184025

**Published:** 2020-09-10

**Authors:** Reza Hosseinpourpia, Stergios Adamopoulos, Thomas Walther, Valeri Naydenov

**Affiliations:** 1 Department of Forestry and Wood Technology, Linnaeus University, Lückligs Plats 1, 35195 Växjö, Sweden; reza.hosseinpourpia@lnu.se; 2 IKEA Industry AB, 215 32 Malmö, Sweden; thomas.walther@inter.ikea.com; 3 SunPine AB, 941 43 Piteå, Sweden; valeri.naydenov@sunpine.se

**Keywords:** tall oil bio-refinery, water repellents, cellulosic fibers, dynamic water vapor sorption, interfiber bonding, thickness swelling, internal bond strength, high-density fiberboard

## Abstract

This study investigates the effect of renewable formulations based on tall oil bio-refinery products on the water vapor sorption and interfiber strength of cellulosic fibers as well as on the properties of high-density fiberboard (HDF) panels. The results obtained for HDF prepared using renewable formulations were compared to the results for HDF obtained using conventional synthetic paraffin wax (hydrowax), which is the hydrophobic agent currently utilized by the industry. Four tall oil distillation products (TODPs) with different levels of fatty and rosin acids were used for preparing the hydrophobic formulations with furfuryl alcohol as an organic solvent. According to determinations with an automated vapor sorption apparatus, the formulations had a similar effect with hydrowax on the sorption behavior of natural fibers. Unlike to hydrowax treatment, the ultimate tensile strength of cellulosic paper-sheets treated with the formulations remained unchanged or significantly increased. At the standard addition load of 1% (wt/wt dry fibers) of the formulations, HDF panels showed comparable and only in one case, e.g., TODP3-based formulation, slightly higher thickness swelling (24 h) than those with hydrowax. The best performing formulation (TODP2-based) in terms of tensile strength of paper sheets did not significantly change the mechanical properties of HDF panels in both standard climate and high humid conditions. Promising results at the standard and humid climate conditions were obtained for HDF panels manufactured with higher TODP2-based formulation amounts (3–5%) and reduced melamine-urea-formaldehyde resin content (10–12% instead of 14%, wt dry resin/wt dry fibers).

## 1. Introduction

For fiberboard manufacturing, refined lignocellulosic fibers are combined with formaldehyde-based synthetic resins such as urea and melamine-urea formaldehyde, and joined together under heat and pressure to form panels. Generally, fiberboards can be classified depending on their density. Low-density fiberboard (LDF) has a density less than 400 kg·m^−3^, medium-density fiberboard (MDF) has a density range of 400~800 kg·m^−3^ and high-density fiberboard (HDF) has densities between 800 and 1100 kg·m^−3^. Like solid wood and other wood-based composites, fiberboard is a hygroscopic material; therefore, its moisture content depends on the surrounding relative humidity and temperature [1]. In addition, urea-formaldehyde is a hydrophilic resin, and even the cured resin is susceptible to hydrolysis under appropriate conditions of elevated temperature and humidity [2]. During processing, finishing and in service, fiberboard panels are exposed to extensive changes in ambient relative humidity, which directly affect the properties of final products, i.e., thickness swelling and linear expansion, surface smoothness, thermal conductivity, stiffness and strength and formaldehyde emissions. Petroleum-based paraffin wax is the main hydrophobic agent for wood-based panels, which can be applied purely in a melted form or as water-based dispersions [3,4]. In the latter form, paraffin wax, so called hydrowax, can also be added to the resin, and thus injected into the blowline [5]. As outlined by Roffael and co-workers [6], a small amount of hydrowax, (0.1 to 1% based on the dry mass of fibers), is efficient to improve the water repellent properties of the panels with no adverse effect on their bond quality and strength properties. In recent years, sustainability of the wood panel industry has drawn more attention due to the heavy dependence on petroleum-derived chemicals, especially adhesives, used in the production [7]. Surprisingly, the efforts for finding industrially viable alternatives to hydrowax additives based on renewable materials has received little attention. Thus, replacing hydrowax with renewable-based additives opens up for the industry to improve their sustainability profile, as a single fiberboard factory alone utilizes annually from ca 600 to more than 1000 tones paraffin wax.

Tall oil is a by-product of Kraft pulping with an average global production of 1.2 million tn/year [8]. The removal of tall oil from the black liquor in the Kraft recovery process presents many advantages while it also has a commercial value [9]. Tall oil is mainly composed of fatty acids (e.g., oleic acid, stearic acid, margaric acid and palmitic acid), rosin acids (e.g., abietic acid, dehydroabietic acid, neoabietic acid, palustric acid, pimaric acid and isopimaric acid) and small amounts of unsaponifiable matter (e.g., sterols, waxes and hydrocarbon) [10]. The percentage of each component, however, depends on several parameters such as wood species, geographical origin, age of trees, wood storage and pulping process [9,10,11]. Crude tall oil can be refined by vacuum distillation to various commercial products such as tall oil rosin acid (25–30% of the total) together with light oil (10–15%), tall oil fatty acid (20–40%), distilled tall oil (approx. 10%) and tall oil pitch (20–30%) [12]. The refined fractions are mainly used for the production of drying oils, soaps, lubricants, linoleum, paints, varnishes, biofuel and energy. 

Tall oil-based solutions have been widely applied previously to improve the hydrophobicity and durability of wood and wood-based products [13,14,15,16,17,18,19,20,21]. Although these studies have mostly shown positive results, the main problems that limit the use of tall oil as a wood protection agent are the large amount needed and its tendency to exude from the wood [22]. On the other hand, studies on the hydrophobization of cellulosic fibers and fiber-based composites with tall oil are quite scarce. Kulomaa and colleagues showed that the modification of cellulosic fibers with tall oil fatty acids enhanced the thermal stability and decreased the water vapor transmission rates [23]. Application of tall oil fatty acid in manufacturing MDF panels improved their dimensional stability, although thickness swelling values were still inferior when compared to those provided by hydrowax [24]. Most recently, Hosseinpourpia and co-workers utilized crude tall oil, tall oil fatty acid and distilled tall oil to improve the hydrophobicity of cellulosic fibers [25]. The authors reported that the equilibrium moisture content of cellulosic fibers was substantially reduced after treatment with these tall oil types. The reduction of moisture sorption was more pronounced at higher relative humidity levels (75–95%) as compared with hydrowax. Crude tall oil and distilled tall oil provided stiffer and more hydrophobic matrices than hydrowax and tall oil fatty acid, which increased the interfiber bonding in both dry and wet state as revealed by tensile strength tests of treated cellulosic papers. It seems that the effects can be attributed to several factors that relate not only to the chemical composition of the tall oils, but also to the strength of the matrices they form and the interfacial adhesion among fibers and matrices. 

The studies mentioned above suggest that a good potential exists for using tall oil-based additives providing water repellent function in various fiber-based products. The distillation process of crude tall oil yields a number of products of varying degree of refining. This study was undertaken with an aim to evaluate the potential of these specific products as alternatives to petroleum-based paraffin wax in HDF manufacturing.

## 2. Materials and Methods 

### 2.1. Materials

Four different tall oil distillation products (TODPs) derived in a distillation process of crude tall oil, denoted TODP1-4 throughout this study, were kindly provided by SunPine AB, Piteå, Sweden. The TODPs are characterized with varying degrees of refining and hence different physical appearances. Thus, at standard laboratory conditions, TODP1 and 2 were dark in color with a viscous paste-like appearance. In contrast, TODP3 (oily liquid) and TODP4 (solid) products were transparent and lighter in color in comparison to the TODP1 and 2. 

Furfuryl alcohol was purchased from Sigma Aldrich, Saint Louis, MO, USA. A commercial Whatman cellulose filter paper-sheet (Whatman, 1443-090) with diameter of 142 mm was purchased from Sigma-Aldrich (Seelze, Germany). Refined fibers obtained from thermo-mechanical-pulping (TMP) of Scots pine (*Pinus sylvestris* L.) wood were purchased from IHD—Institut für Holztechnologie Dresden, Germany. Melamine-urea-formaldehyde (MUF) with a solid content of 66% was kindly provided by IKEA’s HDF factory in Orla, Poland. Ammonium sulphate (wt 40%) was used as a resin hardener. A commercially available hydrowax (Sasol hydrowax 138) with a solid content of 58–62% and pH~9 was supplied by Sasol Germany GmbH, Hamburg, Germany.

### 2.2. Preparation of TODP-Based Formulations and Treatment of Cellulosic Paper-Sheets

The TODPs were tested as hydrophobic substances in the form of solutions in furfuryl alcohol as shown in Table 1. The TODPs were initially heated in an oil bath with respect to their melting temperature for 30 min. Then, the preheated organic solvent at 65 °C, i.e., furfuryl alcohol, was slowly added to the melted TODPs by mechanical stirring to reach similar solid content levels to the extent possible (Table 1). That was due to the different and largely unknown solubility of the TODPs at this point of the research. The mixtures were stirred at 50 °C for 30 min and then taken out of the oil bath for a direct application, i.e., impregnation of paper sheets or HDF manufacturing.

The TODP-based formulations were then analyzed with respect to their effect on mechanical and water vapor sorption properties of cellulose paper-sheets, and the results were compared with those obtained with petroleum-based hydrowax, as described previously [25]. As shown in Table 1, three TODPs had quite similar solid content while one TODP was applied as received in liquid form. Under these boundaries, a preliminary evaluation of their hydrophobic potential was possible in this study. Briefly, commercial Whatman cellulose filter paper-sheets were impregnated with same amount of TODP-based formulations (Table 1) for 5 min under ambient conditions to ensure maximum retention and uniform coverage of all cellulosic fibers with each formulation type. An identical procedure was applied to treat paper-sheets with petroleum-based hydrowax. For comparison, untreated control paper-sheets were also submerged in demineralized water. Finally, the paper-sheets were dried in an oven at 70 °C for 72 h.

### 2.3. Fiberboard Manufacturing

HDF panels were manufactured by standardized procedures that simulated industrial production in the laboratory. Two HDF panels with 3 mm thickness and average density of 860 kg·m^−3^ were produced for each combination, according to Table 2, to test the performance of the hydrophobic formulations. For HDF manufacturing, dry fibers (approximately 2% moisture content) were first mixed with the hydrophobic agents (wt hydrophobic agent/wt dry fiber), for 3 min and then the fibers were resinated with different resin loads (wt dry resin/wt dry fiber) for an additional 5 min, according to Table 2 using a laboratory glue blender (Lödige FM 130D, Paderborn, Germany). Each of the four TODPs was tested as a solution in furfuryl alcohol in a similar amount with paraffin wax (1%) for HDF panels made with MUF resin level of 14%. Preliminary results with paper-sheets showed that TODP2-based formulations showed high tensile strength values in a dry state, which implied a potential of this particular TODP to improve interfiber bonding. Thus, higher amounts of TODP2-based formulations of 3 and 5% were combined with lower MUF resin levels (10–12%) to test the hypothesis of contribution in fiber bonding in HDF panels. 

**Figure 1:**
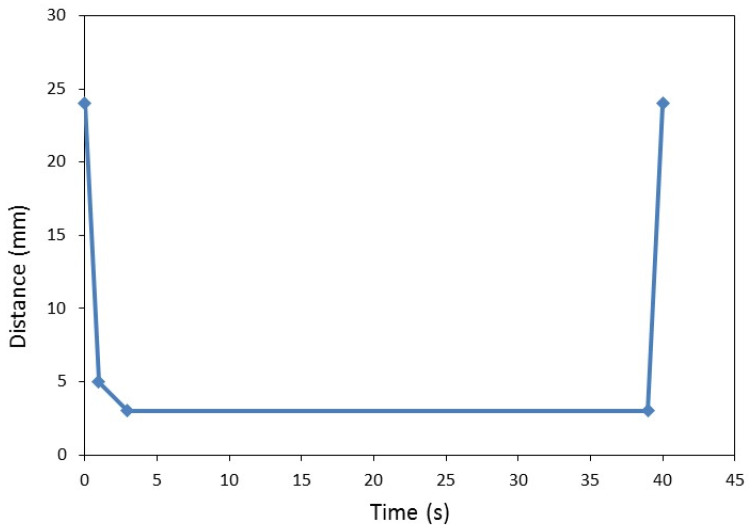
Pressing distance program for HDF production.

The fiber mats were formed by hand (600 × 600 mm^2^) and cold pre-pressed. Hot-pressing was performed at 190 °C and the pressing time was set to 12 s·mm^−1^ using a Joos LAP500OK hydraulic press (Gottfried Joos Maschinenfabrik GmbH & Co. KG, Pfalzgrafenweiler, Germany) according to the pressing distance program shown in Figure 1.

After hot-pressing, the HDF panels were cooled to room temperature, and cut into various test pieces according to the respective standards for evaluating physical and mechanical properties, as described below. Prior to mechanical testing, all samples were conditioned for 14 days in standard and humid climate conditions of 20 °C/65% RH and 28 °C/85%, respectively. The samples for physical testing were only stored in standard condition for a similar period. 

### 2.4. Characterization

#### 2.4.1. Characterization of TODPs

The TODPs were characterized according to their acid value and rosin acid content through acid-base titration method using a Mettler-Toledo T70 titrator (Mettler-Toledo AB, Stockholm, Sweden) and a potentiometric electrode for waterless samples according to ASTM D 803–15:2020 [26] standard. For acid value determination, the samples were dissolved in ethanol before analysis. For rosin acid content analysis, the samples were dissolved in methanol prior to adding a methyl sulfuric acid solution and refluxing. Free fatty acids were calculated according to PCTM 20:1996 [27] method, based on acid value and rosin acid content.

#### 2.4.2. Properties of Cellulosic Paper-Sheets

##### Tensile Strength 

The tensile strength of treated and untreated paper-sheets was determined with the tensile testing machine Z3 (Thümler GmbH, Nürnberg, Germany) according to BS ISO1924-3 standards, as described previously [25,28]. The distance between the clamps were 50 mm at an elongation rate of 100 mm·min^−1^. The dimensions of the samples (paper strips) were 75 × 15 mm^2^. For each treatment, 10 samples were used in dry measuring conditions.

##### Dynamic Vapor Sorption

The dynamic water vapor sorption behavior of the paper samples was determined using an automated sorption balance apparatus (Q5000 SA, TA Instruments, New Castle, DE, USA), according to Hosseinpourpia and co-workers [25,29]. Approximately 8 mg of oven-dried (at 40 °C for 24 h) treated and untreated paper samples were used for each measurement. The relative humidity (RH) increased from 0 to 90% in step sequences of 15% and then 5% from 90 to 95% RH. The instrument maintained a constant target RH until the mass change in the sample (dm/dt) was <0.01% per minute over a 10 min period. The target RH, actual RH, sample mass and running time were recorded every 30 s during the sorption run. The moisture content of the samples was calculated based on their equilibrium weight at each given RH step throughout the sorption run measured by the sorption balance device using the following Equation (1):

(1) $$EMC=\left(\frac{M_{1}-M_{0}}{M_{0}}\right)\times 100$$

where *M*_1_ is the weight of the sample in equilibrium condition with respective RH (g); *M*_0_ is the dry weight of the sample (g).

#### 2.4.3. Testing of HDF Panel Properties

Two HDF panels were used to evaluate the physical and mechanical properties for each of the hydrophobic systems tested (see Table 2). Static bending tests were performed to determine the moduli of rupture (MOR) and elasticity (MOE) of the HDF panels (7 samples per panel, *n* = 14) according to EN 310:1993 [30] using a Zwick 100 testing machine (Zwick GmbH & Co. KG, Ulm, Germany). Rectangular samples measuring 150 × 50 mm^2^ were tested using a span of 100 mm and a speed of 15 mm·min^−1^. The portion between 10 and 40% maximum load was considered for measuring the MOE, as described previously [31]. Internal bond (IB) strength test or tensile strength perpendicular to the surface of panels was conducted following EN 319:1993 [32]. Five samples measuring 50 × 50 mm^2^ per HDF panel (*n* = 10) were effectively bonded with a hot-melt glue and the tension test was performed using the Zwick machine. A loading speed of 1 mm·min^−1^ was used for testing. 

The water-related properties of the HDF panels were determined using 50 × 50 mm^2^ samples immersed in water. Thickness swelling (TS) was assessed after 2 and 24 h of water soaking, while water uptake (WU) was determined after 24h immersion in water. TS (%) was evaluated by the difference between the final and initial thickness, according to EN 317:1993 [33] using 5 samples per panel (*n* = 10).

### 2.5. Statistical Analysis

One-way analysis of variance (ANOVA) was performed on the results at a 95% confidence interval (*p* < 0.05) by means of SPSS version 25.0 statistical software package (IBM Corp., Armonk, NY, USA). The statistical differences between values were assessed by Tukey’s honestly significant difference (HSD) test.

## 3. Results and discussion

### 3.1. Chemical Composition of TODPs

Table 3 lists the general compositions of TODPs utilized within this study. Crude tall oil and the various refining products are complex mixtures. Thus, it is widely accepted to characterize them by acid value parameter, which in turn can be related to the free fatty and rosin acid component groups comprising the material [34]. Each of these two groups is comprised of a relatively large number of individual components. In addition to the acidic part of the material, there is a non-acidic group of components often referred to as neutrals (remaining part up to 100 wt %). As shown from the data listed in Table 3, the TODP1 and 2 can be considered as very similar, i.e., having comparable fatty-and rosin acid contents and relatively low acid values in comparison to TODP3 and 4. However, TODP1 and 2 products are obtained from different points of the refining process, hence with different degree of refining as well as with different commercial value. In the spirit of the exploratory character of the study, the TODP3 and 4 were selected as to represent highly acidic materials, i.e., the neutral part of the material is only few weight percent and TODP3 is fatty acid dominated whereas TOPD4 is rosin acid dominated. 

### 3.2. Properties of Cellulosic Paper-Sheets

**Figure 2:**
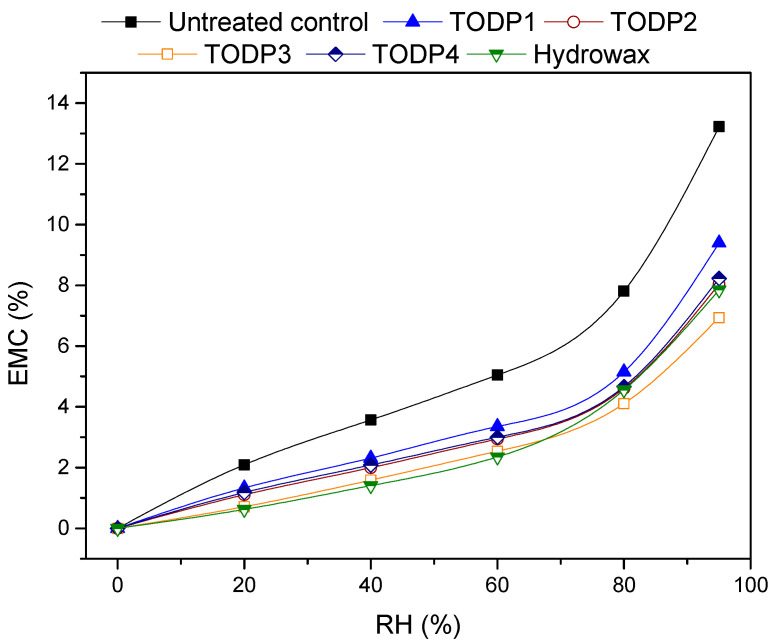
Equilibrium moisture content (EMC) of paper samples treated with hydrowax and with TODP1-, TODP2-, TODP3- and TODP4-based formulations during the adsorption process.

The results on water vapor sorption indicated considerable changes in equilibrium moisture content (EMC) of paper-sheets after treatment with TODP-based formulations and hydrowax during adsorption from 0% to 95% relative humidity (RH) (Figure 2). Treatment with TODP-based formulations caused a noticeable reduction in EMC of cellulose paper-sheets, which confirms their function as hydrophobic agents. The slightly different sorption behavior of paper-sheets treated with the formulations can be attributed to the existence of various amounts of rosin and fatty acids in their composition. Except the TODP1 treated paper, the other papers exhibited comparable or lower EMC values in comparison with the hydrowax treated one, particularly at higher RH levels. However, the differences between TODP1 and hydrowax treated papers were small. Hydrowax-treated paper displayed a low EMC value in the RH range of 0 to 60% and then considerably increased from 80% to 95%. The paper treated with a TODP3-based formulation showed a greater reduction in moisture adsorption as compared with the other treated papers. This can be explained by a considerably higher fatty acid content of this TODP in comparison with the others, as shown in Table 3. According to Hosseinpourpia et al. [25], no chemical reaction occurred between cellulosic fibers and tall oil fractions rich in fatty acids and thus the reduction was attributed to the masking of available sorption sites in fibers’ cell walls by hydrophobic polymers. This particular water-repellent effect that is associated with a nonbond between the cell wall and the hydrophobic agent is similar to that imparted by hydrowax [35,36]. The presence of a considerable amount of rosin acids in the structure of TODP4 should have synergistically created a more effective hydrophobic barrier for the cellulosic fibers, particularly at RHs above 75%. It was shown previously [25] that rosin acids contained in TODPs can react with hydroxyl groups in cellulosic fibers, and this fact can cause an additional hydrophobic effect. The differences in the sorption behavior of the papers treated with the TODP1 and TODP2 formulations might be attributed to the slightly higher rosin content in TODP2, which contributes to a lower EMC in the TODP2 paper-treated sample or the differences could be related to the neutral matrix present for these formulations.

**Figure 3:**
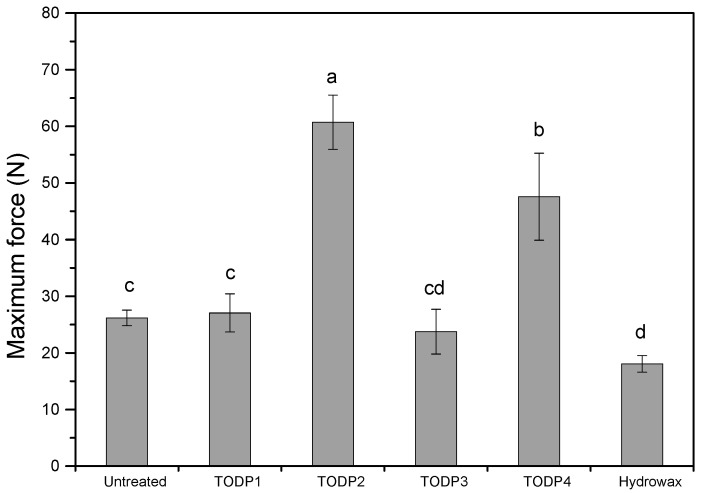
Ultimate tensile strength of paper-sheets treated with TODP-based formulations and hydrowax under dry measuring conditions. Values labeled with a different letter are statistically different (ANOVA and Tukey’s HSD test) at an error probability of α = 0.05. Error bars represent standard deviations.

Paper-sheet samples were assessed by finite-span tensile testing to evaluate the bonding quality between the cellulosic fibers as a function of treatments with TODP-based formulations and hydrowax. The untreated control paper-sheets showed an ultimate tensile strength of ~26 N under the dry measuring condition (Figure 3). The paper-sheets treated with TODP1-and TODP3-based formulations showed comparable ultimate tensile strength as untreated control paper samples, while the TODP2 and TODF4 ones significantly increased the strength of the paper-sheets (ANOVA, α = 0.05). A statistically significant reduction in the ultimate tensile strength of paper-sheet samples was observed after treatment with hydrowax, which was ~19 N (ANOVA, α = 0.05). These results are in accordance with previous studies [25,37,38]. As explained previously, strength reduction of hydrowax-treated paper samples can be attributed to a) allocation of a considerable amount of low to medium molecular weight fractions of hydrophobic hydrowax between the fibers, which may hinder the equal distribution of the applied mechanical stresses around the fibers, and thus reduce the interfiber bonding strength, and b) formation of hydrowax matrices with poor mechanical strength that encompassed the cellulosic fibers [37,39]. A look at the data presented in Figure 3 reveals that paper-sheets treated with TODP2- and TODP4-based formulations demonstrate superior bonding strength. One possible explanation could be the fact that these two formulations have highest rosin acid contents, where the rosin acids interpenetrate into the fiber cell walls and create a stiff matrix that in turn is embedding the fibers. Following this line of reasoning, the positive effect of rosin acids might have been offset by the existence of a considerable amount of unsaturated fatty acids in the TODP3-based formulation that resulted in a matrix with low mechanical strength instead, but nevertheless comparable to untreated paper sheet. Somewhat puzzling is the value obtained for TODP1-treated paper-sheet, where a comparable ultimate tensile strength with untreated control paper-sheet is observed. The behavior of TODP1 is in contrast and difficult to explain in view of the value obtained for TODP2 (highest ultimate strength among all treatments with differences statistically significant (ANOVA and Tukey’s HSD test, α = 0.05), especially when considered together with compositional data (Table 3). Based on the general compositional data (Table 3), the TODP1 and 2 are similar but the two formulations affect in significantly different manner the mechanical strength of the paper sheets. At this stage of the study, the difference is most likely related to the presence of a substantial amount of neutral components. Keeping in mind that the TODP1 and 2 are obtained at different stages of the refining process, it is most likely that the individual neutral components differ for the two materials and/or have different abundance, which will be a subject for further investigations. The current understanding is that the TODP2-based formulation is capable of providing much stiffer matrices than TODP1. The differences in bonding strength however cannot be related to rosin acids alone, which is obvious from the results obtained for paper-sheets treated with TODP4 formulation, i.e., the formulation with highest rosin content (Figure 3). The exceptional performance of TODP2 to create water-repellent formulations comparable to hydrowax that also fortify significantly the fiber network, can be particularly useful in enhancing the mechanical properties of fiber-based products such as HDF.

### 3.3. Properties of HDF Panels 

**Figure 4:**
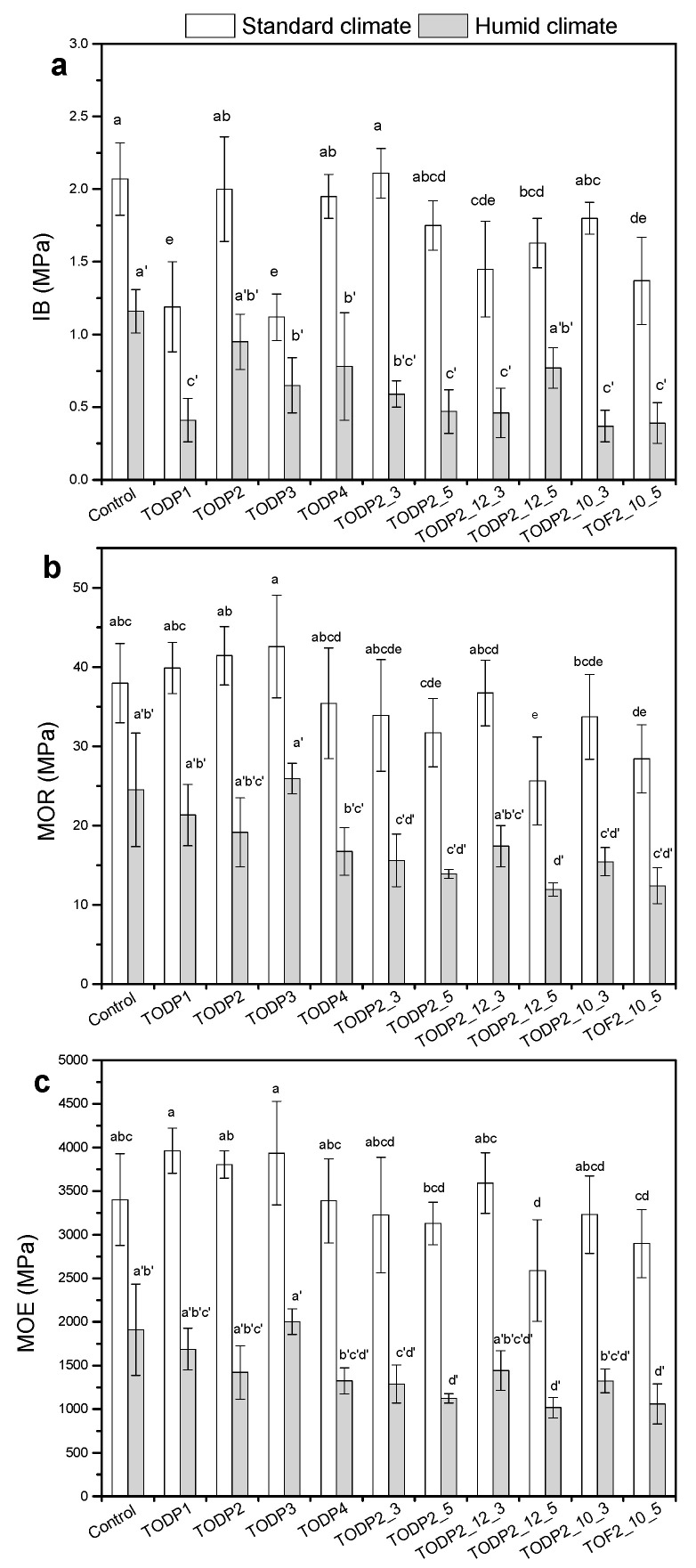
(**a**) Internal bond strength (IB), (**b**) modulus of rupture (MOR) and (**c**) modulus of elasticity (MOE) of the HDF panels under standard and humid climate conditions. Values labeled with a different letter are statistically different (ANOVA and Tukey’s HSD test) at an error probability of α = 0.05. Error bars represent standard deviations.

Figure 4a–c shows the mechanical properties of HDF panels together with details of statistical comparisons (ANOVA and Tukey’s HSD test, α = 0.05) as a function of hydrophobic agent and MUF resin contents under standard and humid climate conditions. The internal bond (IB) strength is an important quality parameter of the bonding between fibers with the aid of a resin and indicates the adhesion quality of the panel in the direction perpendicular to its plane. Under standard climate conditions and with a typical MUF amount of 14% for laboratory scale HDF panels, the panels manufactured with TODP2-and TODP4-based formulations showed comparable IB strength to the controls (Figure 4a). These results are in agreement with the ultimate tensile strength results in cellulose paper-sheets (Figure 3), and indicate the potential of these two formulations to enhance the interfiber bonding quality in a simple fiber network (paper) or at least not disturb the adhesive bonding in more complex fiber networks of a fiber-based product like HDF. HDF panels with TODP1-and TODP3-based formulations had a similar IB performance but their values were significantly lower than the controls for the standard climate conditions. In humid conditions and typical MUF resin load, only the HDF panels manufactured with TODP2-based formulation had a similar IB strength with the controls. Increasing the TODP2 load from 1% to 3% and 5% was not found to change significantly the IB strength for HDF panels with typical MUF resin amount under standard climate conditions. This is an interesting finding since the addition of paraffin at higher amounts than 1% adversely affects the bond quality and reduces the IB strength of fiberboards [6,40]. Such an increase in the amount of the TODP2-formulation did not favor the IB strength under humid conditions. When the MUF resin load was reduced from 14% to 12% and 10%, only one combination of TODP2-formulation and MUF resin could achieve the IB strength of controls (14% MUF resin; 1% hydrowax) per standard (3% and 10%, respectively) and humid climate conditions (5% and 12%, respectively). It seems that the MUF resin load is still a prevailing parameter for an adequate IB strength under both climate conditions. However, more research is needed in this respect since TODP2-formulation seemed to have some contribution in the adhesion of HDF panels manufactured with lower MUF contents, but the results did not follow any particular pattern.

Using TODP-based formulations with 1% loading did not change significantly the static bending properties (MOR, MOE) in HDF panels manufactured with the typical MUF resin content (14%) as compared with controls for both climate conditions (Figure 4b,c). That was also true when TODP2-based formulation was added at the higher loads of 3% and 5% but only in the case of standard climate conditions. In general, for both climate conditions and as compared to controls, the moduli of rupture and elasticity were found to significantly decrease at the higher amount of TODP2-based formulation (5%) in HDF panels produced with lower MUF resin content (10 and 12%). Another general observation was that static bending properties of HDF panels manufactured with TODP-based formulation were less affected than IB strength.

The reduction in the mechanical strength of wood-based panels under humid conditions is an expected result [2,41,42], which can be attributed to the relative brittleness and inability of cured resin to be plasticized through the induced stress by water molecules at a high relative humidity [43,44]. This may lead to failure in interfiber connection, and thus reduce the mechanical strength of the HDF panels. It should be noted that testing of mechanical properties of wood-based panels according to standards is performed under standard climate conditions. Thus, the results of the present study under humid conditions can only serve for comparisons of the effects of TODP-based formulations against those obtained with hydrowax. Such comparisons are quite useful since HDF panels can be produced in Europe to be exported and used later in Asia, where climate conditions are different, i.e., higher temperatures and RHs. 

**Figure 5:**
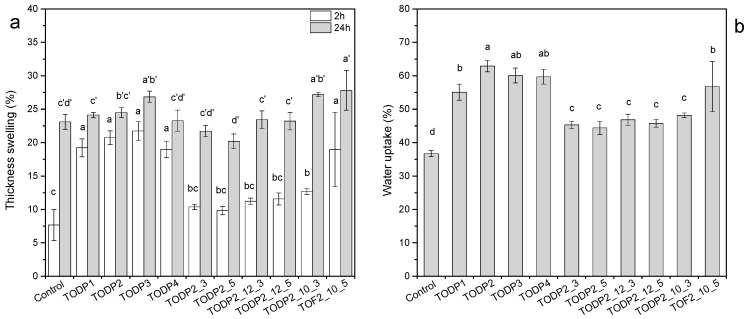
(**a**) The 2 h and 24 h thickness swelling (TS), (**b**) 24 h water uptake of the HDF panels immersed in water. Values labeled with a different letter are statistically different (ANOVA and Tukey’s HSD test) at an error probability of α = 0.05. Error bars represent standard deviations.

Thickness swelling (TS) and associated statistical analysis (ANOVA and Tukey’s HSD test, α = 0.05) of the HDF panels manufactured with different TODP-based formulations is presented in Figure 5a. The control panel showed TS values of 8% and 23% measured after 2 and 24 h, respectively. TS swelling increased significantly in panels with all TODP-based formulations added at 1% and typical MUF amount (14%) after 2h measuring time. TODP2-based formulations added at higher amounts (3 and 5%) performed better in this respect and had comparable TS values with the control (hydrowax) with the exception of panels manufactured with the lowest MUF content of 10%. However, the impact of TODP-based hydrophobic agents on TS of HDF panels was more evident after 24 h of immersion in water. These results showed an excellent water-repellent effect for most of the TODP-based formulations. Again, the two TODP2-based formulations with the lowest MUF content showed significantly higher TS than the control panel as well as the TODP3-based formulation. These findings mostly agree with the hydrophobic effects of the TODP-based formulations in the cellulose papers (Figure 2). However, the superior hydrophobic performance of TODP3 noted in the papers was not repeated in HDF, and this particular TODP could not impart adequate dimensional stability like hydrowax for the control panel. The water uptake (WU) of all HDF panels with TODP-based hydrophobic agents significantly increased after 24 h immersion in water (Figure 5b). Although, WU significantly decreased by increasing the TODP2 content in typical and reduced MUF resin loads, values differed statistically as compared to the control HDF. The water absorption of fiberboards is mainly attributed to the ability of fibers to absorb water [45]. Addition of TODP-based formulations may have created a hydrophobic corset around fibers, and in some cases chemical reactions between their constituents and cell walls’ hydroxyl groups might have occurred. Since the high-molecular-weight polymers of the TODP-based formulations were deposited on the fibers’ surfaces, smaller fractions might have penetrated into the cell walls of the fibers. As a result, the interaction of fibers with water molecules should have been lower in the case of panels with TODP-based formulations than the control panel with hydrowax. This could explain the fact of higher WU in those panels but lower thickness swelling (24 h). These results are in agreement with a previous study that used amino-alkyl siloxane to improve the hydrophobicity of particleboards [2]. However, the high WU is not a limiting factor, as no threshold value is defined in the EN 317:1993 [33] standard, whereas the thickness swelling values are the critical ones.

## 4. Conclusions

This study investigated whether four products derived in a distillation process of crude tall oil could be used as sustainable raw materials for preparing water repellents for HDF panels. TODP-based formulations in an organic solvent (furfuryl alcohol) were thus compared with hydrowax that is used today by the industry, first in cellulose papers and then in HDF panels. All formulations proved to be as effective as hydrowax in reducing the moisture sorption of papers while at the same time they provided stiffer matrices for the cellulosic fiber network and considerably increased the ultimate tensile strength. All TODP-based formulations at 1% load level performed equally to hydrowax in terms of thickness swelling (24 h) of HDF with 14% MUF resin, thus verifying their hydrophobic potential. Most formulations did not reduce the mechanical properties of the panels as compared with the control, especially under the standard climate conditions, while formulations with TODP2 and TODP4 performed better in this respect. In fact, TODP2-based formulation proved to be superior as HDF panels had similar mechanical properties with the control for both standard and humid conditions. The overall results implied that TODP2 could also perform well in solutions with higher load levels (3 and 5%) without negatively affecting the mechanical properties of panels like hydrowax. Encouraging results were also obtained in combinations of TODP2-based solution with lower MUF resin amount (10–12%). However, the interaction of TODP2-formulation with MUF resin with regards to curing performance and adhesion of HDF requires more research. In conclusion, the effectiveness of TODPs as water repellents for HDF seems to depend on their chemical composition and the mechanical behavior of matrices they create within the fiber network. Thus, ensuring a standard quality of appropriate TODPs from the distillation process should be necessary. Future research should focus on preparing more sustainable TODP-based hydrophobic formulations without the need of an organic solvent, i.e., by using appropriate emulsifiers. Evaluating their effects on the release of formaldehyde and volatile organic compounds from HDF would be also useful. 

## Figures and Tables

**Table 1:** Melting temperature of tall oil distillation products (TODPs) and concentration of the TODP-based formulations in furfuryl alcohol.

TODPs	Melting Temperature (°C)	Solid Content of TODP-Based Formulations (%)
TODP1	65	17
TODP2	70	25
TODP3 *	-	-
TODP4	90	18

* TODP3 was provided in a liquid form and used as received.

**Table 2:** Load level of hydrophobic agents and melamine-urea-formaldehyde (MUF) resin for high-density fiberboard (HDF) production.

Formulations	Hydrophobic Agent Load (%)	MUF Resin Load (%)
Control (Sasol Wax)	1	14
TODP1	1	14
TODP2	1	14
TODP3	1	14
TODF4	1	14
TODP2_3	3	14
TODP2_5	5	14
TODP2_12_3	3	12
TODP2_12_5	5	12
TODP2_10_3	3	10
TODP2_10_5	5	10

**Table 3:** Chemical composition of tall oil distillation products (TODPs).

TODPs	Acid Value (mg KOH/g)	Rosin Acid Content (wt %)	Unsaturated Fatty Acid Content (wt %)
TODP1	61.9	27.1	5.8
TODP2	64.1	30.0	4.3
TODP3	183.9	20.2	73.7
TODP4	175.0	89.8	4.2
